# A Lipidomic Analysis Reveals Dynamic Changes of Polar Lipids for Oil Biosynthesis During Cotyledon Development in *Perilla frutescens*

**DOI:** 10.3390/plants15010119

**Published:** 2026-01-01

**Authors:** Xiaoxiao Liu, Jiudong Zhang, Weijun Xu, Xichun Du, Deng Yang, Lingling Xu, Shuangyu Zhang, Tianpeng Gao

**Affiliations:** 1 School of Biological and Environmental Engineering, Xi’an University, Xi’an 710065, China; xiaoxiaoliulg@163.com (X.L.);; 2 State Key Laboratory of Grassland Agro-Ecosystems, College of Ecology, Lanzhou University, Lanzhou 730000, China; 3 School of Arts and Design, Xi’an University, Xi’an 710065, China; 4 Engineering Center for Pollution Control and Ecological Restoration in Mining of Gansu Province, Lanzhou City University, Lanzhou 730070, China

**Keywords:** perilla, cotyledon, polar lipid, triacylglycerol

## Abstract

Perilla (*Perilla frutescens*) is an important oilseed crop valued for its rich content of nutraceutical compounds and polyunsaturated fatty acids. While triacylglycerol biosynthesis has been studied, the role of polar lipids during seed development remains poorly characterized. Here, we performed a comprehensive lipidomic analysis of polar lipids in developing perilla seeds across three key stages. A total of 147 molecular species from 10 polar lipid classes were identified. Phosphatidylcholine and phosphatidylethanolamine were the predominant phospholipids, and both decreased markedly during development, with phosphatidylcholine showing the most significant reduction. In contrast, lysophosphatidic acid increased substantially by 62.4%. Conversely, the galactolipids monolactodiacylglycerol and digalactosyldiacylglycerol showed a decline in perilla during cotyledon development. Additionally, the unsaturation index of most polar lipids decreased during development. These variation characteristics of polar lipids during growth and development may suggest an adaptive strategy for oil accumulation in perilla.

## 1. Introduction

*Perilla frutescens* L. Britt., commonly known as Perilla, is an annual herbaceous plant belonging to the Lamiaceae family [1]. It is an economically important oil crop widely cultivated in East Asia for its high-value seeds. These seeds are rich in polyunsaturated fatty acids, particularly α-linolenic (18:3) acids, which can account for up to 60% of the total fatty acids in the seed oil [2]. In contrast to conventional oilseed crops like rapeseed, where the embryo is the primary site for triacylglycerols (TAGs) accumulation, the cotyledons of perilla serve as the main location for both lipid biosynthesis and storage. This unique metabolic profile, characterized by both a distinctive fatty acid composition and a specific tissue-level organization of lipid metabolism, positions perilla as a valuable model for understanding natural variation in oilseed biology. However, despite its economic and nutritional importance, a comprehensive, molecular-level description of the dynamic lipid landscape within its developing cotyledons remains lacking.

Glycerolipids, comprising polar membrane lipids and neutral storage lipids, are fundamental structural and metabolic components of plant cell membranes. Their biosynthesis involves coordinated pathways across the plastid and the endoplasmic reticulum (ER). In the plastidic pathway, two sequential acylations of glycerol 3-phosphate (G3P) produce phosphatidic acid (PA) [3,4]. The product of PA is dephosphorylated by lipid phosphate phosphatase γ, LPPε1, and LPPε2, or converted to cytidine diphosphate-diacylglycerol (CDP-DAG) by CDP-DAG synthases CDS4 and CDS5 for PG synthesis or formation of phospholipids [5,6]. Phosphatidylcholine (PC) and phosphatidylethanolamine (PE) are synthesized via analogous reaction steps. Choline and ethanolamine are first phosphorylated by choline kinase and ethanolamine kinase, respectively, resulting in phosphocholine and phosphoethanolamine. These activated intermediates are then attached to the DAG backbone to produce PC and PE [7]. The production of phosphatidylglycerol (PG) and phosphatidylinositol (PI) begins with PA, which is converted to CDP-DAG by CDP-DAG synthase (CDS). Phosphatidylglycerophosphate synthases (PGPSs) subsequently utilize CDP-DAG to generate phosphatidylglycerophosphate (PGP), which is then dephosphorylated by PGP phosphatase (PGPP) to yield PG [8]. PI is synthesized by phosphatidylinositol synthase (PIS). Phosphatidylserine (PS) is produced from PE through a base-exchange reaction, catalyzed by PS synthase (PSS), where serine substitutes for ethanolamine. PS can, in turn, be decarboxylated by PS decarboxylase (PSD) to regenerate PE [9]. The biosynthesis of monogalactosyldiacylglycerol (MGDG) and digalactosyldiacylglycerol (DGDG) involves the galactosylation of DAG. This process is initiated by MGDG synthases (MGDs) to produce MGDG, followed by a second galactosylation catalyzed by DGDG synthases (DGDs) to form DGDG [10].

Triacylglycerol (TAG) is the primary form of storage lipid accumulated in plant mature seeds [11], serving as a reserve of carbon and energy during germination and early seedling growth [12]. The main pathways are known for TAG assembly, the acyl-CoA-dependent Kennedy pathway, and the acyl-CoA-independent PDAT pathway [13]. TAG biosynthesis is metabolically interconnected with that of polar lipids. First, both pathways share PA as a common precursor. PA is a central intermediate in lipid metabolism. For TAG synthesis, it is dephosphorylated by phosphatidic acid phosphohydrolase to produce DAG, which is then acylated by diacylglycerol acyltransferases to form TAG [14]. Simultaneously, PA is utilized for the synthesis of phospholipids, such as PC and PE, via the CDP-DAG pathway. Second, DAG represents another critical metabolic branch point. The DAG produced from PA can be directed toward TAG formation, and PC can undergo remodeling to generate DAG molecules. The DAG can then re-enter the TAG biosynthesis pathway. Third, key enzymes, such as glycerol-3-phosphate acyltransferases (GPATs) and diacylglycerol acyltransferases (DGATs), demonstrate overlapping roles that link TAG and polar lipid biosynthesis pathways. In summary, the TAG biosynthetic network in developing perilla cotyledons is hypothesized to be profoundly intertwined with polar lipid metabolism. This integration may occur through shared precursors (PA, DAG), enzyme substrate specificities, and lipid remodeling processes that coordinately regulate both pathways to balance membrane integrity with massive oil accumulation during seed development. Understanding these metabolic intersections is essential for designing targeted engineering strategies aimed at improving the yield and nutritional quality of perilla seed oil. A simplified schematic diagram summarizing the major glycerolipid and glycerophospholipid biosynthetic pathways is provided in Supplementary Figure S1.

Lipidomics is a branch of metabolomics that can reveal the complete molecular information of lipids in biological samples at a holistic level [15]. It is a rapidly advancing field in health, nutrition, and biomedicine, with broad applications in areas such as nutritional science, disease prediction, and the development of targeted therapies. In plant biology, lipidomics has been recently applied to study oilseed and cereal crops. For example, Xia et al. employed lipidomics to investigate the mechanisms underlying the dynamic accumulation of fatty acids and lipid species in walnut kernels [16]. Lipidomics was also employed to analyze and compare polar lipids in the endosperms of oat and wheat varieties with different oil contents at various developmental stages [17]. In hickory, lipidomics has enabled the identification of 67 TAGS, 27 DAGs, 33 PCs, and 76 PEs [18]. Despite these advances, a comprehensive lipidomic profile of developing perilla seeds remains unreported.

In this study, we performed the lipidomic analysis using liquid chromatography-mass spectrometry (LCMS) to characterize the polar lipid profiles of perilla cotyledons across three key developmental stages. The specific objectives were: (1) to systematically identify and quantify polar lipid molecular species in developing perilla cotyledons; (2) to analyze the temporal changes in polar lipid classes and their unsaturation indices during seed maturation; and (3) to analyze the correlation patterns among lipid classes to infer metabolic coordination. We hypothesized that the unique oil accumulation phenotype in perilla is underpinned by a distinct program of polar lipid remodeling. This study provides the first detailed lipidomic atlas of developing perilla cotyledons, establishing an essential foundation for future targeted investigations into the regulatory mechanisms governing high-value oil production in this nutritionally important crop.

## 2. Results

### 2.1. Changes in Total Lipid and Polar Lipid Contents During Cotyledon Development

**Figure 1:**
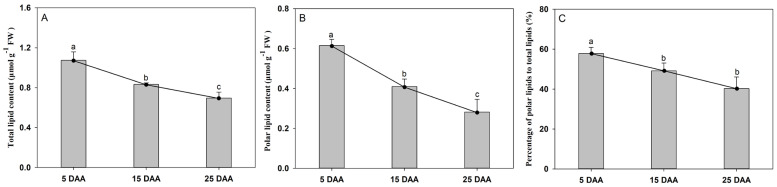
Lipid contents in the developing cotyledon of perilla: (**A**) total lipid content; (**B**) polar lipid content; and (**C**) relative contents of polar lipid to total lipids of cotyledon. DAA indicates days after anthesis. The data are presented as mean ± SD of three biological replicates. The bars followed by different letters are significantly different according to the LSD-test (*p* < 0.05).

To assess the dynamic changes in total lipid content and polar lipid turnover during the development of perilla cotyledons, samples were collected at the early (five days after anthesis, 5 DAA), middle (15 DAA), and late (25 DAA) stages and subjected to quantitative analysis (Figure S2). Both total lipid and polar lipid contents (expressed as μmol per g fresh weight, FW) declined steadily across the three stages, decreasing by 35.1% and 54.1%, respectively, from 5 to 25 DAA (Figure 1A,B). Notably, the observed decline in total lipid content on a fresh weight basis, while seemingly at odds with the well-established accumulation of storage TAGs during seed maturation, likely reflects a profound metabolic reprogramming. The proportion of polar lipids relative to total lipids also decreased continuously during development, showing a reduction of 30.4% 25 DAA compared to the 5 DAA level (Figure 1C). In plants, the levels of polar and neutral lipids reflect the allocation of photosynthetic products. Our results suggest that during early cotyledon development, a larger proportion of photosynthetic products is allocated toward polar lipid synthesis to support membrane biogenesis. As development proceeds, however, lipid-bound carbon is increasingly channeled into neutral lipid accumulation, indicating a metabolic shift from membrane construction to storage deposition.

### 2.2. Polar Lipid Species and Dynamics During Cotyledon Development

**Figure 2:**
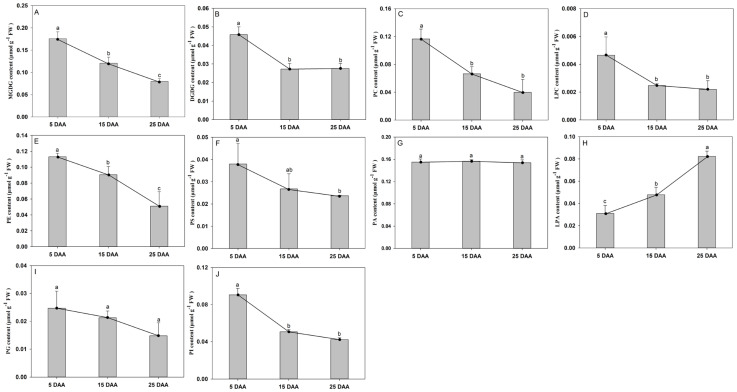
Polar lipid contents in the developing cotyledon of perilla: (**A**) MGDG; (**B**) DGDG; (**C**) PC; (**D**) LPC; (**E**) PE; (**F**) PS; (**G**) PA; (**H**) LPA; (**I**) PG; and (**J**) PI contents. MGDG, monogalactosyldiacylglycerol; DGDG, digalactosyldiacylglycerol; PC, phosphatidylcholine; LPC, lysophosphatidylcholine; PE, phosphatidylethanolamine; PS, phosphatidylserine; PA, phosphatidic acid; LPA, lysophosphatidic acid; PG, phosphatidylglycerol; PI, phosphatidylinositol. DAA indicates days after anthesis. The data are presented as mean ± SD of three biological replicates. The bars followed by different letters are significantly different according to the LSD-test (*p* < 0.05).

Through lipidomic analysis, the dynamically changing patterns of polar lipids in perilla seeds have been clarified. A total of 147 polar lipids were identified, belonging to 10 categories, including 16 MGDGs, 17 DGDGs, 33 PCs, 13 PEs, 16 PGs, 22 PIs, 11 PSs, 8 PAs, 5 LPCs, and 6 LPAs. The contents of MGDG, PC, PE, PG, and PI collectively decreased during seed development. Among these, PC showed the most significant decline, decreasing by 65.8% between 5 and 25 DAA, followed by PE (54.8%), MGDG (54.6%), PI (53.3%), and PG (40.1%) (Figure 2). The contents of DGDG and PS decreased from 5 to 15 DAA and then remained relatively stable from 15 to 25 DAA (Figure 2B,F). The levels of LPC and PA remained stable throughout the developmental stages. In contrast, the content of LPA showed an increasing trend during cotyledon development (especially between 15 and 25 DAA), with the content at 25 DAA increasing by 62.4% compared to that at 5 DAA (Figure 2H).

To further elucidate differences in lipid composition during cotyledon development, a cluster analysis was performed on the relative abundance of polar lipid categories in perilla at different stages. The clustering method used was Ward.D2, and the analysis reflected the fold change of the standard deviation relative to the mean for each lipid category. The resulting heatmap revealed that PE, PG, DGDG, LPC, PS, PI, MGDG, and PC were present at higher levels during early cotyledon development and gradually decreased or remained stable as development proceeded. In contrast, LPA content showed an increasing trend during cotyledon development (Figure S3).

### 2.3. Polar Lipid Species and Dynamic Changes During Cotyledon Development

**Figure 3:**
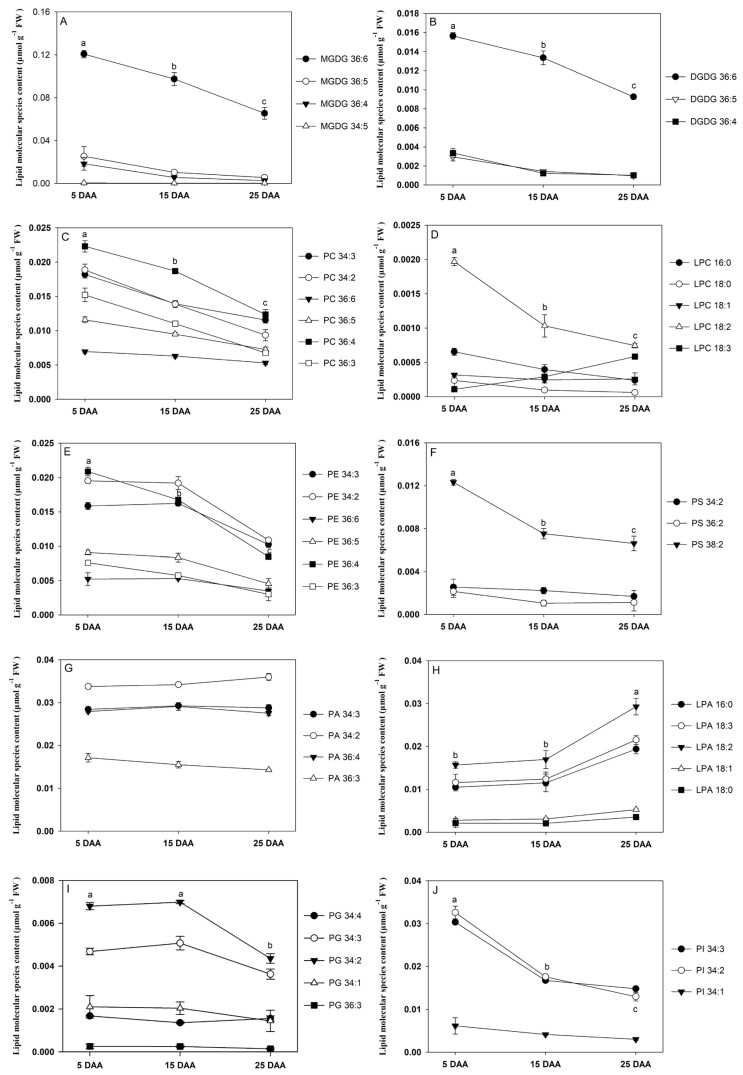
Major polar lipid molecular species in the developing cotyledon of perilla: (**A**) monogalactosyldiacylglycerol (MGDG); (**B**) digalactosyldiacylglycerol (DGDG); (**C**) phosphatidylcholine (PC); (**D**) lysophosphatidylcholine (LPC); (**E**) phosphatidylethanolamine (PE); (**F**) phosphatidylserine (PS); (**G**) phosphatidic acid (PA); (**H**) lysophosphatidic acid (LPA); (**I**) phosphatidylglycerol (PG); and (**J**) phosphatidylinositol (PI). DAA indicates days after anthesis. The data are presented as mean ± SD of three biological replicates. The bars followed by different letters are significantly different according to the LSD-test (*p* < 0.05).

MGDG and DGDG are glycolipids, which are primary membrane lipids in the thylakoid. The initial development of the cotyledon is dependent on photosynthesis, during which significant alterations in polar lipid species were observed. For MGDG, the main glycerolipid species in perilla cotyledon were C36:6, followed by C36:5 and C36:4, all of which declined during development. Compared to 5 DAA, the contents of C36:6, C36:5, and C36:4 were significantly decreased by 45.8%, 78.3%, and 85.4%, respectively, 25 DAA (Figure 3A). Low-abundance glycerolipid species such as C34:5 did not change significantly, while other MGDG species, including C37:3, C36:3, C35:6, C34:3, C34:2, and C32:4, also declined. The contents of C34:6 and C35:3 increased early in development, but decreased between 15 and 25 DAA (Figure S4A). For DGDG, the dominant species were C36:6, C36:5 and C36:4, all of which decreased over time. Compared to 5 DAA, the contents of C36:6, C36:5, and C36:4 decreased by 41.0%, 67.1%, and 69.6%, respectively, 25 DAA (Figure 3B). Other detected species, including C37:3, C36:3, C36:2, C35:3, C34:3, C34:2, and C34:1, were present at low levels in the perilla cotyledon at all developmental stages.

Phospholipids play crucial roles in membrane metabolism and TAG biosynthesis. Lysophospholipids are intermediates of phospholipid metabolism. The main PA species in perilla cotyledon was C36:4. The content of C36:4 decreased by 44.4% between 5 and 25 DAA. Similarly, C34:2, C34:3, C36:3, C36:5 and C36:6 showed a decreasing trend during cotyledon development (Figure 3C). The other PC species identified were C40:3, C38:5, C38:4, C38:3, C38:2, C37:5, C37:4, C37:3, C37:2, C36:2, C36:1, C35:5, C35:4, C35:3, C35:2, C35:1, C34:4, C34:1, C34:0, C33:3, C33:2, C33:1, C33:0, C32:1, and C32:0 with a decrease trend, and C42:3 and C42:2 remain stable during all development stage (Figure S4C).

The dominant LPC species in perilla cotyledon were C18:2 and C16:0. The C18:2 content was high in early cotyledon development, and then gradually decreased. Compared to 5 DAA, the concentration of C18:2 was markedly decreased by 62.2% 25 DAA (Figure 3D). C16:0 was also decreased during the cotyledon development stage. The contents of C18:3, C18:1, and C18:0 were low and maintained stability (Figure S4H).

The dominant PE species were C36:4, C34:2, and C34:3. The content of C36:4, C34:2 and C34:3 decreased by 59.4%, 44.1%, and 35.8%, from 5 to 25 DAA during cotyledon development of perilla (Figure 3E). In addition, the other PE species identified were C37:1, C36:6, C36:5, C36:4, C36:3, C36:2, C36:1, C35:3, C35:2, C34:1, and C33:2, with a low content in perilla and were all decreasing with development (Figure S4F).

The dominant PS species was C38:2. Compared with 5 DAA, the content of C38:2 was dramatically decreased by 46.2% 25 DAA (Figure 3F). The other PS species were C38:3, C38:1, C36:5, C36:4, C36:3, C36:2, C36:1, C34:3, C34:2 and C34:1; they were all reduced between 5 and 25 DAA (Figure S4G).

The main PA species in perilla cotyledon were C34:2, C36:4 and C34:3. The C34:2 was stable 5 and 15 DAA and increased 25 DAA. The C36:4 and C34:3 were stable 5 and 15 DAA, and decreased 25 DAA (Figure 3G). The content of C36:3 decreased as the perilla developed. The other identified PA species were C36:6, C36:5, C36:3, C36:2 and C34:4 with low content.

The main LPA species was C18:2. The C18:2 content was low in the early and middle cotyledon development (5 and 15 DAA), and greatly increased in the late cotyledon development (25 DAA). Compared to 5 DAA, the C18:2 content increased by 46.4% 25 DAA (Figure 3H). The content of C18:3 and C16:0 was similar to that of C18:2 at all development stages. The other LPA species were C18:1, C18:0, C17:0, and 16:0. They were all increased in the cotyledon development stages (Figure S4E).

The main PG species was C34:2, which increased slightly from 5 to 15 DAA during the cotyledon development in perilla but declined greatly in the late cotyledon development (15 to 25 DAA). Compared to 5 DAA, the content of C34:2 decreased by 36.0% 25 DAA (Figure 3I). Other PG species identified included C36:5, C36:3, C36:2, C35:6, C35:3, C35:2, C34:5, C34:4, C34:3, C34:1, C34:0, C33:2, C33:0, C32:1, and C32:0, with C34:3 showing a trend similar to that of C34:2.

The main PI species in the cotyledon of perilla were C34:3 and C34:2, both of which decreased sharply during early development (5 to 15 DAA) and continued to decline through the last stage (15 to 25 DAA) (Figure 3J). Other PI species, including C36:6, C36:5, C36:4, C36:3, C36:2, C36:1, C35:5, C35:4, C35:3, C35:2, C35:1, 35:0. C34:1, C34:0, C33:3, C33:2, C33:1, C33:0, C32:1, and C32:0, exhibited similar declining trends across development (Figure S4J).

Analysis of the correlation coefficient values of polar lipid classes during cotyledon development showed that MGDG and DGDG were positively correlated with each other. Most phospholipids, including PC, PE, PG, PS, and PI, showed significant positive correlations among themselves, forming a closely associated network. In contrast, LPA displayed broad and significant negative correlations with several lipid classes, most notably with MGDG, PG, and PI. PA showed no significant correlation with any of the other major lipid classes measured (Figure S5).

### 2.4. Differential Analysis of Polar Lipid Species During Cotyledon Development

Principal component analysis (PCA) is a commonly used method in metabolomics. From the score plot, we can observe the overall differences among different samples. We performed PCA, and the results showed that the two principal components of PC1 and PC2 distinguished perilla at different stages of cotyledon development. There was a significant difference in polar lipid species at different cotyledon development stages. The two PCA axes encompassed 77.4% of the observed total variance by lipid species (Figure S6), showing that the three stages of cotyledon development of perilla had certain distinctions in the first two principal component dimensions.

**Figure 4:**
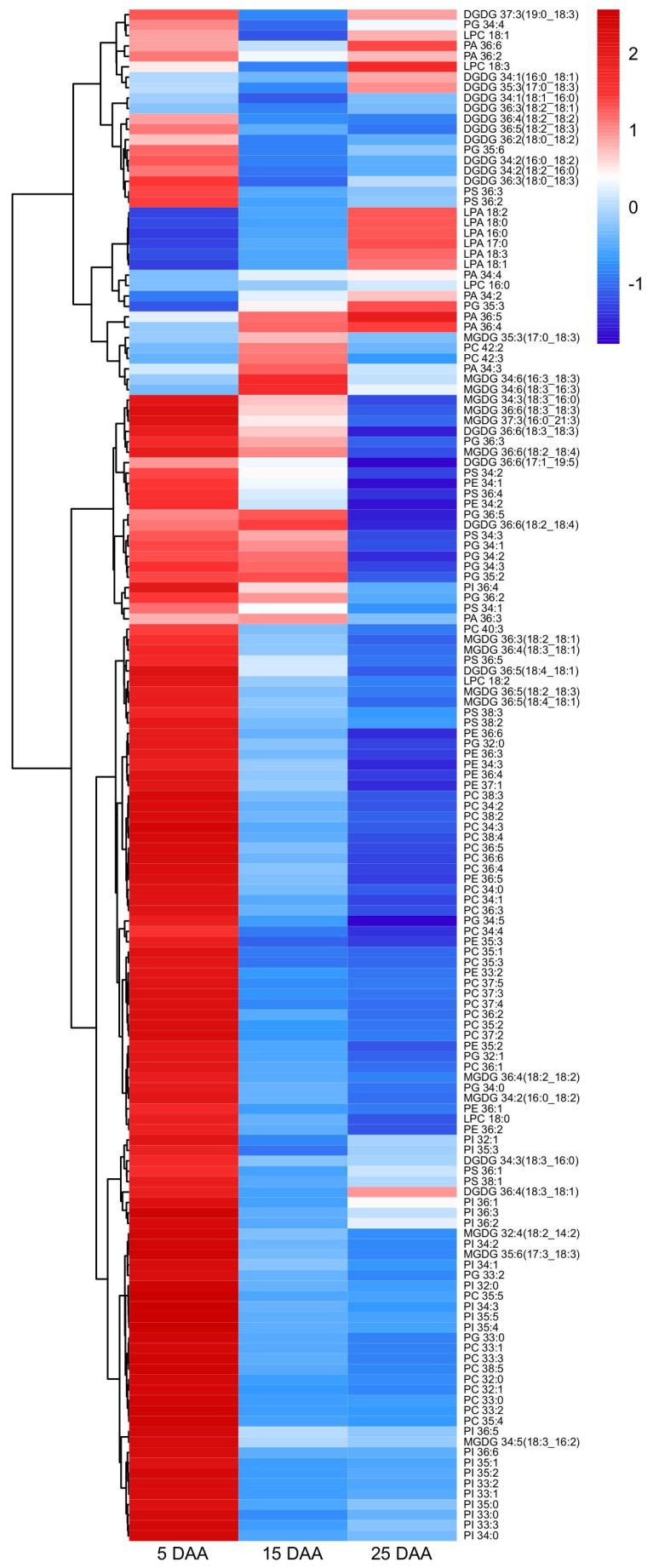
Heatmap of the contents of lipid molecule species at three developmental stages in the cotyledon of perilla. Each row represents an individual lipid species, with abundance values normalized using Z-scores. Hierarchical clustering was performed based on the Euclidean distance measure and the Ward.D2 method for linkage analysis. DAA indicates days after anthesis.

To further compare and analyze the changes in lipid species at the three stages of perilla cotyledon development, heatmaps were conducted for the polar lipids of perilla cotyledon. Due to the significant differences in the content of various metabolites, we first standardize all the metabolites. The changes in polar lipid content across the three stages are shown in Figure 4. Polar lipid species with a higher abundance in the cotyledon at each stage of perilla were mainly concentrated in the early development stage (5 DAA), from MGDG 34:3 (18:3_16:0) to PI 34:0. Furthermore, three subgroups were identified in these species in the development stages. In the first subgroup (from MGDG 34:3 (18:3_16:0) to PA 36:3), polar lipid species were mainly accumulated 5 and 15 DAA and showed a low level 25 DAA. The second subgroup, from PC 40:3 to PE 36:2, showed a high level 5 DAA, a lower level 15 DAA, and an even lower level 25 DAA. The third group, from PI 32:1 to PI 34:0, had high content 5 DAA, and a lower abundance at 15 DAA and 25 DAA of perilla. Apart from these lipids, the remaining types of lipids were divided into two subgroups, which concentrated in the higher half of Figure 4, from DGDG 37:3 (19:0_18:3) to MGDG 34:6 (18:3_16:3). The lipid species in the first subgroup, from DGDG 37:3 (19:0_18:3) to PS 36:2, had higher content of polar lipid 5 and 25 DAA, whereas they had lower abundance 15 DAA. The second group, from LPA 18:2 to MGDG 34:6 (18:3_16:3), mainly showed a high level 15 and 25 DAA, and a lower level 5 DAA in perilla.

### 2.5. Analysis of the Unsaturation Degree of Polar Lipid During Cotyledon Development

**Figure 5:**
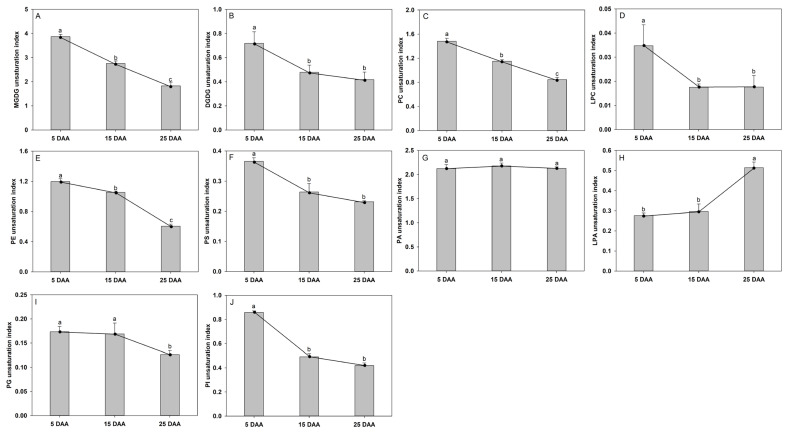
Unsaturation index of polar lipid molecular species in the developing cotyledon of perilla: (**A**) monogalactosyldiacylglycerol (MGDG); (**B**) digalactosyldiacylglycerol (DGDG); (**C**) phosphatidylcholine (PC); (**D**) lysophosphatidylcholine (LPC); (**E**) phosphatidylethanolamine (PE); (**F**) phosphatidylserine (PS); (**G**) phosphatidic acid (PA); (**H**) lysophosphatidic acid (LPA); (**I**) phosphatidylglycerol (PG); and (**J**) phosphatidylinositol (PI). DAA indicates days after anthesis. The data are presented as mean ± SD of three biological replicates. The bars followed by different letters are significantly different according to the LSD-test (*p* < 0.05).

Analysis of the unsaturation degree of polar lipids provides insight into lipid transport dynamics. In this study, we calculated the unsaturation index of polar lipid to compare changes in acyl group unsaturation across developmental stages (Figure 5). Compared to 5 DAA, the significant decreases in unsaturation index were identified in MGDG, PC, PE, and PI 25 DAA, which decreased by 52.7%, 42.9%, 49.5%, and 51.3%, respectively. The unsaturation index of DGDG and PS showed the same trend of alteration; there was a slightly downward trend from 5 to 15 DAA, and it remained stable from 15 to 25 DAA. The general trend of unsaturation indices of LPC and LPA remained stable 5 and 15 DAA and increased from 15 to 25 DAA. The unsaturation indices of PA and PG remained stable from 5 to 15 DAA and decreased from 15 to 25 DAA.

## 3. Discussion

Oilseeds are a crucial source of diversified nutraceuticals for humans. Perilla is a well-known oilseed crop valued for its edible oil and medicinal products [19]. Understanding the dynamic changes in oil metabolism during perilla cotyledon development is crucial for elucidating the metabolic adaptations that support oil accumulation. While most research on perilla has focused on the features of TAG biosynthesis [14,20,21], the biosynthesis of TAG is not an isolated pathway but is closely linked with polar lipid metabolism [17]. They share common precursors and enzymatic steps. Therefore, characterizing polar lipid profiles can provide insights into the composition and dynamics of the broader lipid metabolic network during development. Moreover, understanding the synthesis and turnover of polar lipids is key to elucidating the molecular mechanisms governing lipid accumulation and overall oil yield in perilla seeds.

Our study revealed a progressive decline in both the absolute content of polar lipids and their proportion relative to total lipids in developing perilla cotyledons 5, 15, and 25 DAA. The total extractable lipid content also decreased on a fresh weight basis. This observation, consistent with findings in other oil-rich seeds such as oat [17], underscores a fundamental metabolic reprogramming rather than a contradiction. The declining proportion of polar lipids directly correlates with the re-partitioning of carbon towards massive neutral lipid (TAG) accumulation during seed maturation [22]. Since polar lipids and TAG are the major components of total lipids, their contents are necessarily inversely related, but this quantitative inverse relationship does not carry direct biological significance. A more meaningful biological indicator is the polar lipid content by mass, which reflects the proportion of photosynthetic products allocated to total polar lipids. Similar to reports in walnut, oil content was low at the early development stage and increased as the cotyledon developed [23]. This observed negative correlation between polar lipid proportion and total lipid accumulation aligns with findings in other oilseeds like oat, where a higher carbon allocation to TAG appeared to be coupled with a relative reduction in polar lipid investment [24]. As maturation progresses, metabolism shifts toward storage lipid production. The data are consistent with two non-exclusive possibilities: First, de novo synthesized fatty acids may be increasingly channeled toward TAG assembly. Second, pre-existing polar lipids may undergo remodeling, potentially providing acyl groups (like α-linolenic acid) for TAG synthesis, a mechanism noted in high-oil oat varieties [17]. However, this does not prove that the changes in polar lipids contribute to the deposition of TAG. Studies have shown that the accumulation of polar lipids may not be essential for the accumulation of lipids in oat endosperm [17]. Therefore, the turnover of polar lipids may be the most important dynamic process.

In this study, polar lipids during cotyledon development consisted of 10 classes of glycolipids and phospholipids. The main glycolipids were MGDG and DGDG, the predominant phospholipids were PE and PC, and the major lysophospholipids were LPA and LPC (Figure 2). At the early development stage, embryos of perilla were present in a gelatinous and watery state (5 DAA). Accompanying the development, the embryos began to be filled with a white cotyledon (Figure S2). The observed progressive decline in the content of major polar membrane lipids, including MGDG, PC, PE, PG, and PI, during perilla cotyledon development coincides with the physiological process of endosperm degeneration [25]. Our results show a marked reduction in these components, with PC experiencing the most dramatic decrease, followed by PE, MGDG, PI, and PG. PC can be hydrolyzed or converted to other lipid classes via many pathways [26]. Phospholipase C (PLC) and phospholipase D (PLD) can convert PC to DAG and PA, and generate TAG and LPC [25,27]. This pattern of decline is consistent with the possibility that the large-scale degradation of endosperm cellular structures, which are rich in plastidial (MGDG) and extra-plastidial (PC, PE) membranes, could serve as a source of fatty acids and carbon skeletons. Concurrently, the metabolic priority of the seed shifts from membrane biogenesis to storage accumulation. The pronounced decline in PC is consistent with its potential role not only as a structural lipid but also as a key precursor in the Kennedy and PC-DAG pathways for TAG biosynthesis [28,29]. The differential rates of reduction among these lipids may reflect distinct metabolic fluxes during cotyledon expansion.

In contrast, the stabilization of certain lipids and the rise of LPA highlight the balance between degradation and maintenance of essential cellular components. The initial decline and subsequent stabilization of DGDG and PS after 15 DAA may relate to their roles in maintaining structural integrity in the developing cotyledons. PA is an important substrate for both membrane phospholipids and TAG biosynthesis [30]. Combined with the embryonic development state, PA may be utilized more for phospholipids synthesis before 5 DAA to support cell growth and membrane maintenance, and at the late development stage, the PA was used for the assembly of TAGs, contributing to oil accumulation in perilla. These results were similar to the formation of oil bodies in walnut seeds [23]. Importantly, the stable levels of LPC and PA throughout all stages suggest tightly regulated turnover, consistent with their roles as metabolic intermediates and signaling molecules, respectively [31,32]. The most striking change later in development was the significant accumulation of LPA (increasing 62.4% from 5 DAA to 25 DAA), particularly between 15 and 25 DAA. The increase is consistent with an increased flux through the Kennedy pathway, where LPA production could temporarily exceed its consumption by lysophosphatidic acid acyltransferases (LPAAT), potentially indicating active de novo TAG synthesis [33]. Alternatively, it may relate to enhanced phospholipase-mediated activity during seed maturation. Thus, lipid remodeling in perilla demonstrates a complex strategy that appears to repurpose membrane lipids for energy storage while preserving specific lipids likely essential for cotyledon development. To further understand the coordination within this remodeling network, we performed a correlation analysis on the levels of different polar lipid classes across the developmental stage (Figure S5). The resulting correlation matrix revealed distinct association patterns that reinforce the inferred metabolic transitions. Strong positive correlations were observed among the major membrane galactolipids (MGDG and DGDG) and between the phospholipids PC and PE, suggesting their coordinated regulation as core membrane components. Importantly, the accumulation of LPA showed a significant negative correlation with several phospholipid classes (PC, PE) that declined substantially over time. This inverse relationship is consistent with a model where the degradation or remodeling of these phospholipid pools is metabolically linked to the production of LPA as part of the Lands cycle [34]. Crucially, evidence from Arabidopsis indicates that LPA derived from membrane PC can be channeled into the Kennedy pathway to support TAG synthesis [35], supporting the notion of an enhanced flux through this pathway for subsequent storage oil assembly.

The development of perilla cotyledons is accompanied by substantial remodeling of polar lipid molecular species, reflecting shifts in metabolic priorities. In this study, a total of 147 molecular species from 10 classes of polar lipids were identified in the developing cotyledon of perilla. As primary components of thylakoid membranes, the glycolipids MGDG and DGDG exhibited pronounced declines in their predominant molecular species. The most abundant MGDG species (C36:6, C36:5, and C36:4) decreased significantly from 5 to 25 DAA. A similar trend was observed in DGDG, where C36:6, C36:5, and C36:4 decreased. The preferential reduction in polyunsaturated species implies large-scale disassembly of photosynthetic membranes as cotyledons transition from autotrophy to storage tissue. Concurrently, dominant species of PC, PE, and PS (such as C36:4 in PC and PE, and C38:2 in PS) showed marked reductions. The decreased content of lysophospholipid LPC (C18:2) suggested active acyl turnover. These results are consistent with the alterations in lipid metabolism reported in germinating soybean seeds, which also showed reduced glycerolipids and phospholipids alongside increased lysophospholipids [36]. In contrast, PA species displayed class-specific behavior, C34:2 remained stable initially but increased by 25 DAA, possibly indicating its role as a precursor for TAG synthesis or lipid signaling [37]. Most strikingly, LPA (C18:2) increased markedly 25 DAA. These patterns collectively are consistent with a metabolic shift from membrane lipid synthesis toward TAG accumulation. It has been reported that high pH increased TAG accumulation but decreased glycolipid and polar lipid in *Chlorella* [38]. Therefore, the decline in glycolipids and most phospholipids could reflect the degradation of organellar membranes and redirection of fatty acids into storage lipids. The stability or rise of specific PA and LPA species might indicate potential regulatory nodes in the lipid metabolic networks [39]. The distinct compositional dynamics also suggest that lipid molecular species could serve as markers for developmental transitions in oil-rich seeds such as perilla.

Analysis of the unsaturation index (UI) of polar lipids during perilla cotyledon development revealed dynamic and class-specific patterns, reflecting active lipid remodeling and shifts in metabolic function. A pronounced decrease in UI was observed in several major lipid classes between 5 and 25 DAA, including MGDG, PC, PE, and PI. This widespread reduction in unsaturation is consistent with the large-scale degradation of highly polyunsaturated membrane lipids and the recycling of their acyl chains into TAG assembly. The decline in UI is consistent with a metabolic shift away from membrane lipid production toward TAG accumulation, which is considered a hallmark of oilseed maturation [36]. These temporal and class-specific trends in unsaturation highlight a coordinated lipid metabolic network. Further research into the expression and activity of desaturases and phospholipases during these stages may clarify whether the unsaturation changes result from reduced modification activity or accelerated lipid turnover. Finally, it should be noted that this study provides a detailed lipidomic landscape of developing perilla cotyledons. While we have included photographic documentation of seed morphology across three stages (Figure S2) to provide essential visual context, future investigations integrating more comprehensive morphometric characterization (cotyledon size, fresh/dry weight), along with physiological and biochemical data, will further solidify the quantitative link between lipid remodeling and structural development.

## 4. Materials and Methods

### 4.1. Plant Materials and Growth Conditions

Perilla plants were cultivated according to a standard agronomic management in the experimental area of Xi’an University, Shaanxi, China. The seedlings were grown in a greenhouse providing natural sunlight, supplemented with artificial lighting (LED panels, 16-h light/8-h dark photoperiod) to ensure consistent light intensity. Temperature and relative humidity were maintained within the ranges of 25–35 °C and 50–70%, respectively, using an automated environmental control system. Fertilizers of N:P:K at a concentration of 130:100:90 kg/ha and organic fertilizer (N + P + K > 5%) were applied before sowing. During the middle of the perilla flowering period, each fully open flower was labeled in the afternoon. With reference to the developmental stages of perilla seeds [40], seeds in this study were collected starting from 5 to 25 DAA, with a 10-day interval between each harvest. Seeds from three different development stages were collected. To ensure sample representativeness, cotyledons from every three plants were thoroughly mixed to form one biological replicate, and three biological replicates were applied for each developmental stage.

### 4.2. Lipid Extraction

Cotyledon samples were weighed immediately after harvest. To minimize phospholipid degradation, the cotyledon-rich tissues were quickly transferred to 400 μL of preheated isopropanol (75 °C) containing 0.01% butylated hydroxytoluene (BHT) in a 15 mL glass tube, using a modified experimental protocol [41]. Each sample was incubated at 75 °C for 18 min to inactivate lipid-hydrolyzing enzymes, and then cooled to room temperature. Samples were stored at −80 °C and then shipped on dry ice to the analytical facility. After inactivation, an extraction solvent consisting of chloroform–methanol–300 mM ammonium acetate (30:41.5:3.5, *v*/*v*/*v*) was added to the samples, which were then incubated at 4 °C with shaking at 1500 rpm for 30 min. After incubation, the samples were centrifuged, and the clarified supernatant was transferred to new tubes. To improve lipid recovery, the tissue pellet was then extracted a second time with fresh extraction solvent (without additional heating/inactivation), following the same incubation and centrifugation procedure. The lipid extracts from both rounds were combined and dried using a high-speed vacuum pump (Genevac, UK). The dried lipid extracts were stored at −80 °C until liquid chromatography mass spectrometry (LC-MS) analysis. The lipidomic data are calculated by using a known molar amount of internal standards, the content of the internal standards, and the weight of the extracted samples to determine the content of each lipid molecule type in the group. Results are expressed as contents per gram fresh weight (μmol/g FW). This approach provides directly comparable quantitative data across all developmental stages.

### 4.3. Liquid Chromatography Mass Spectrometry Analysis

All lipidomics analyses were performed at LipidALL Technologies Company using a Shimadzu Nexera 20AD-HPLC system coupled with a Sciex QTRAP 6500 PLUS mass spectrometer (Sciex, Boston, USA), as previously described [42]. For normal-phase analysis of polar lipids, separation was carried out on a TUP-HB silica column (150 × 2.1 mm inner diameter, 3 μm particle size) under the following conditions: mobile phase A (chloroform–methanol –ammonium hydroxide, 89.5:10:0.5) and mobile phase B (chloroform–methanol–ammonium hydroxide–water, 55:39:0.5:5.5). This NP-HPLC step was employed to separate the total lipid extract into major classes (glycolipids vs. phospholipids) based on the polarity of their head groups, thereby simplifying the mixture for subsequent detailed analysis. For reversed-phase liquid chromatography/mass spectrometry (RP-LC/MS) analysis, lipid detection was performed using a modified reversed-phase (RP)-high-performance liquid chromatography/electrospray ionization/tandem mass spectrometry (HPLC/ESI/MS/MS) method consistent with previous reports [21]. The subsequent RP-HPLC-MS/MS analysis provided fine separation of lipid molecular species within each class based on fatty acyl chain hydrophobicity, enabling definitive identification and absolute quantification via tandem mass spectrometry with isotope-labeled internal standards. Quantification of individual lipid species was performed by referencing added internal standards, including d9-PC32:0 (16:0/16:0), d7-PE33:1 (15:0/18:1), d31-PS (d31-16:0/18:1), d7-PA33:1 (15:0/18:1), d7-PG33:1 (15:0/18:1), d7-LPC18:1, MGDG 34:0, DGDG 36:0 (all purchased from Avanti Polar Lipids, Alabaster, Alabama), as well as LIPID MAPS. Octadecyl phosphatidylinositol (PI) (16:0-PI) was obtained from Echelon Biosciences, Inc. (Salt Lake City, UT, USA) and used together with d7-PI33:1 (15:0/18:1) (purchased from Avanti Polar Lipids) for PI quantification. Free fatty acids were quantified using d31-16:0 (purchased from Thermo Fisher Scientific, Waltham, MA, USA).

The identified lipid molecular species included MGDG, DGDG, PC, LPC, PE, PS, PA, LPA, PG, and PI. The purpose of quantification is to compare the content of each lipid molecular species across different samples. A quality control (QC) approach was adopted to ensure that the data for each molecular species remained comparable throughout the mass spectrometry data acquisition process [43,44]. The quality of the data was assessed using QC analysis by generating a correlation matrix plot of QC samples. In this matrix, the data from two QC samples were log10-transformed and pairwise compared. Each point in the scatter plot below the diagonal represents a lipid species. A tight linear distribution of all points indicates high consistency between the two QC samples. The corresponding correlation coefficient is displayed above the diagonal; a value greater than 0.99 indicates excellent consistency and high data quality.

### 4.4. Statistical Analysis

The lipidomic analysis in this study was carried out with three biological replicates, and the data are expressed as mean ± standard deviation (SD). The principal component analysis (PCA) was performed by the NovoMagic cloud platform (https://magic.novogene.com/), and the cluster heat map analysis was conducted by the Hiplot Pro platform (https://hiplot.com.cn/). ANOVA was performed using SPSS Statistics Analysis System (Version 20.0 for Windows, SPSS, Chicago, IL, USA). The significance of differences between the mean values was assessed using the least significant difference (LSD) test at *p* < 0.05.

## 5. Conclusions

In this study, the lipidomic analysis revealed dynamic changes in polar lipid profiles during perilla seed development. A total of 10 polar lipid classes and 147 molecular species were identified, with significant temporal changes in composition and unsaturation index observed across key developmental stages. The substantial decline in major membrane lipids (PC, PE, and MGDG) alongside the pronounced accumulation of LPA in later stages reflects an extensive reprogramming of polar lipid metabolism. These patterns indicate that the remodeling of polar lipid pools is closely associated with the metabolic network that facilitates oil deposition in perilla cotyledons. Our findings suggest that improving oil production in perilla and other oilseed crops might depend not only on enhancing TAG biosynthetic capacity but also on understanding and potentially modulating the dynamic remodeling of polar lipids. This study provides a detailed lipidomic atlas and valuable insights into the lipid metabolic adaptations underlying high oil accumulation in perilla. Future studies incorporating targeted analysis of neutral lipids (DAG and TAG species), enzyme activities, and isotopic flux are needed to establish direct mechanistic links and to delineate the complete flux network of storage lipid biosynthesis.

## Data Availability

Data are contained within the article and Supplementary Materials.

## Supplementary Materials

The following supporting information can be downloaded at: https://www.mdpi.com/article/10.3390/plants15010119/s1, Figure S1. A simplified schematic diagram of major glycerolipid and glycerophospholipid biosynthetic pathways; Figure S2. Morphological changes of perilla seeds at key developmental stages. A, 5 days after anthesis (5 DAA); B, 15 days after anthesis (15 DAA); C, 25 days after anthesis (25 DAA). Scale bar = 0.5 mm; Figure S3. Heatmap of the polar lipid classes at each developmental stage in cotyledon of perilla. Each row corresponds to a distinct lipid class, and the contents of polar lipids were normalized using Z-scores. Hierarchical clustering was applied based on Euclidean distance measure and Ward.D2 method. PE, phosphatidylethanolamine; LPC, lysophosphatidylcholine; PS, phosphatidylserine; PG, phosphatidylglycerol; DGDG, digalactosyldiacylglycerol; PI, phosphatidylinositol MGDG, monogalactosyldiacylglycerol; PC, phosphatidylcholine; PA, phosphatidic acid; LPA, lysophosphatidic acid; Figure S4. Heatmap of each polar lipid molecule specie type at three developmental stage in cotyledon of perilla. A, monogalactosyldiacylglycerol (MGDG); B, digalactosyldiacylglycerol (DGDG); C, phosphatidylcholine (PC); D, phosphatidic acid (PA); E, lysophosphatidic acid (LPA); F, phosphatidylethanolamine (PE); G, phosphatidylserine (PS); H, lysophosphatidylcholine (LPC); I, phosphatidylglycerol (PG); J, phosphatidylinositol (PI); Figure S5. Correlation matrix (based on the Pearson correlation coefficient) of polar lipid classes in developing cotyledons of perilla. MGDG, monogalactosyldiacylglycerol; DGDG, digalactosyldiacylglycerol; PC, phosphatidylcholine; LPC, lysophosphatidylcholine; PE, phosphatidylethanolamine; PS, phosphatidylserine; PA, phosphatidic acid; LPA, lysophosphatidic acid; PG, phosphatidylglycerol; PI, phosphatidylinositol; Figure S6. Principal component analysis (PCA) score plot of polar lipids classes in developing cotyledon of perilla. DAA indicates days after anthesis.
