# Supplementary material for: A Lipidomic Analysis Reveals Dynamic Changes of Polar Lipids for Oil Biosynthesis During Cotyledon Development in Perilla frutescens
Source: Plants (Basel). 2026 Jan 1;15(1):119. doi: 10.3390/plants15010119 (PMC12787418; doi:10.3390/plants15010119)
Supplement: Supplementary file 1 [file plants-15-00119-s001.zip › plants-4024958-supplementary.pdf]

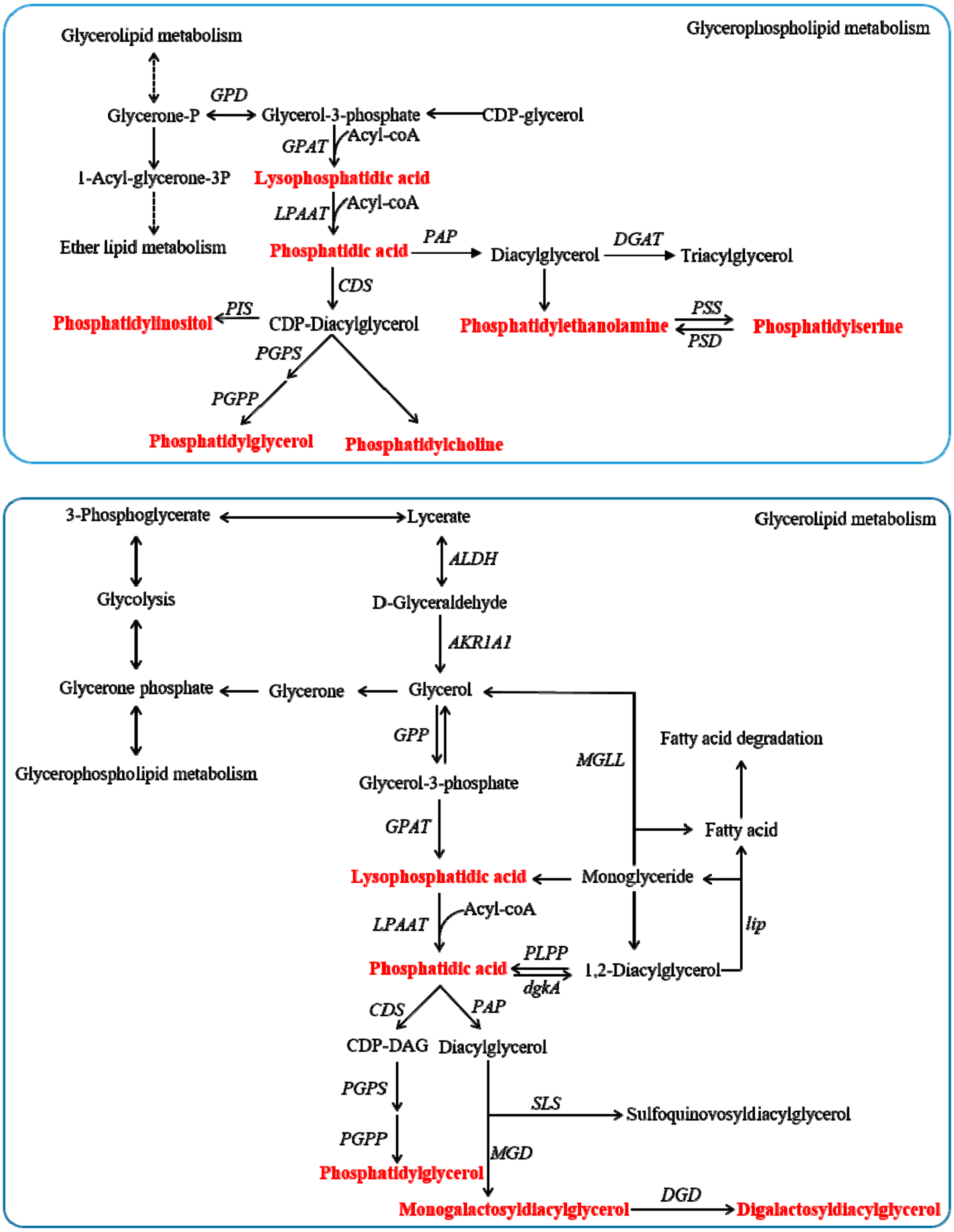

Figure S1. A simplified schematic diagram of major glycerolipid and glycerophospholipid biosynthetic pathways

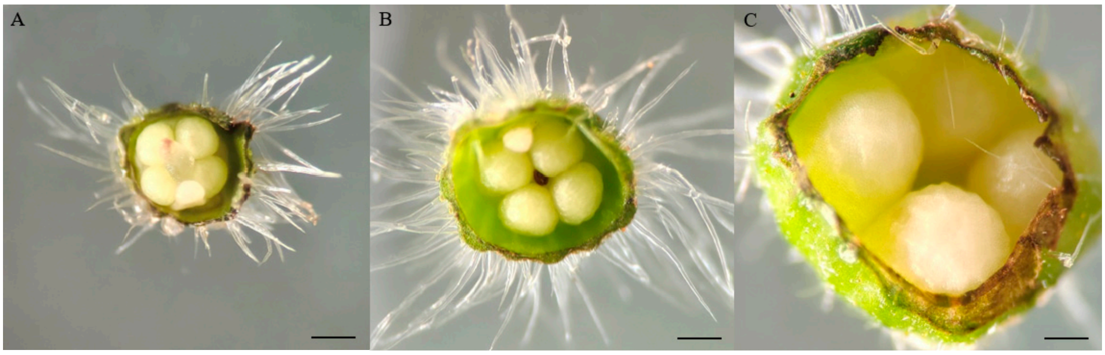

Figure S2. Morphological changes of perilla seeds at key developmental stages. A, 5 days after anthesis (5 DAA); B, 15 days after anthesis (15 DAA); C, 25 days after anthesis (25 DAA). Scale bar = 0.5 mm.

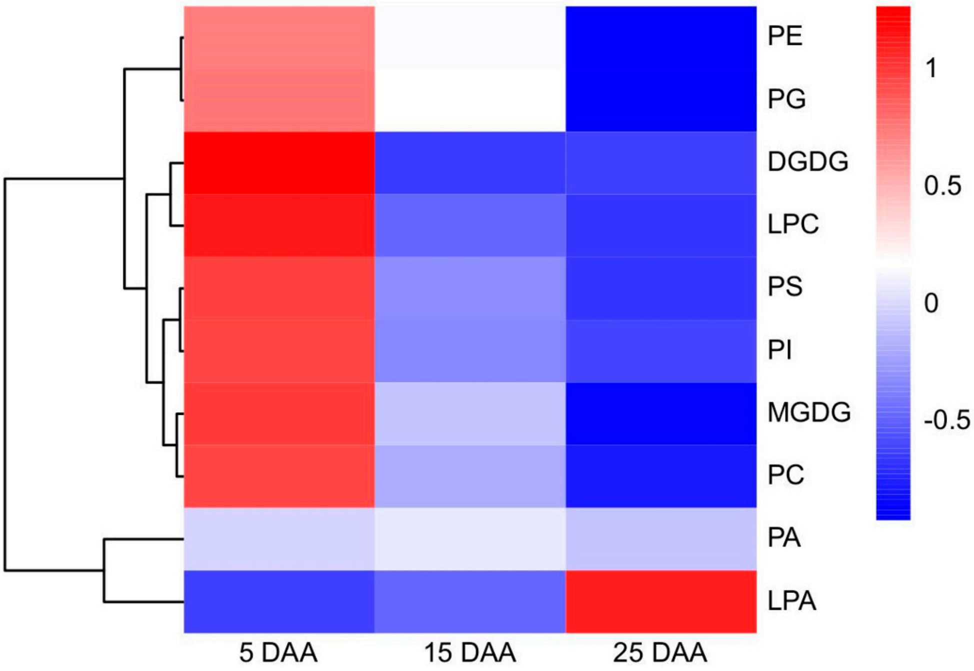

Figure S3. Heatmap of the polar lipid classes at each developmental stage in cotyledon of perilla. Each row corresponds to a distinct lipid class, and the contents of polar lipids were normalized using Z-scores. Hierarchical clustering was applied based on Euclidean distance measure and Ward.D2 method. PE, phosphatidylethanolamine; LPC, lysophosphatidylcholine; PS, phosphatidylserine; PG, phosphatidylglycerol; DGDG, digalactosyldiacylglycerol; PI, phosphatidylinositol MGDG, monogalactosyldiacylglycerol; PC, phosphatidylcholine; PA, phosphatidic acid; LPA, lysophosphatidic acid.

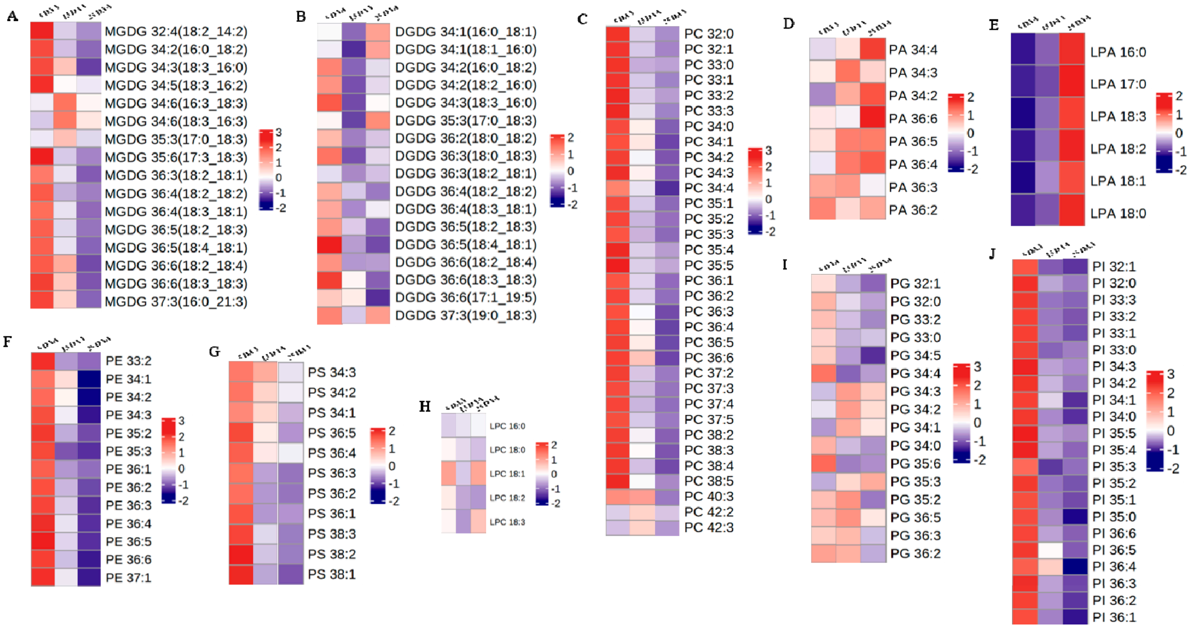

Figure S4. Heatmap of each polar lipid molecule specie type at three developmental stage in cotyledon of perilla. A, monogalactosyldiacylglycerol (MGDG); B, digalactosyldiacylglycerol (DGDG); C, phosphatidylcholine (PC); D, phosphatidic acid (PA); E, lysophosphatidic acid (LPA); F, phosphatidylethanolamine (PE); G, phosphatidylserine (PS); H, lysophosphatidylcholine (LPC); I, phosphatidylglycerol (PG); J, phosphatidylinositol (PI).

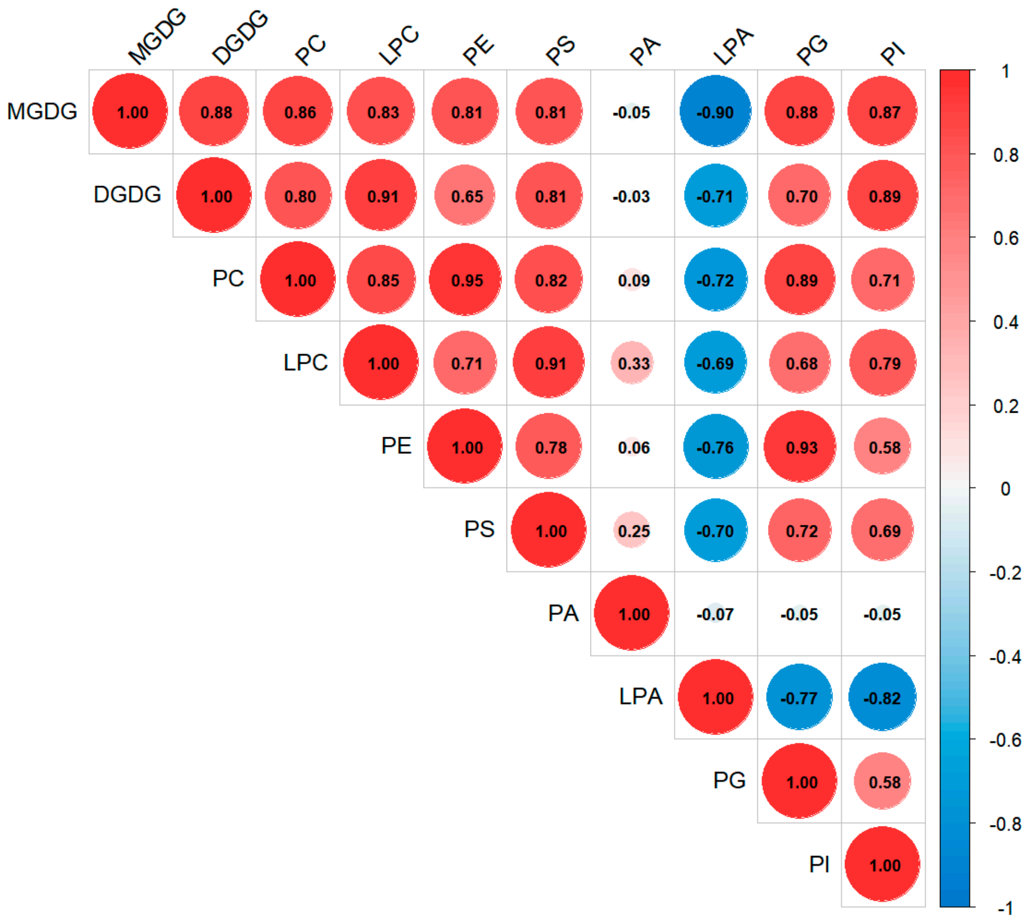

Figure S5. Correlation matrix (based on the Pearson correlation coefficient) of polar lipid classes in developing cotyledons of perilla. MGDG, monogalactosyldiacylglycerol; DGDG, digalactosyldiacylglycerol; PC, phosphatidylcholine; LPC, lysophosphatidylcholine; PE, phosphatidylethanolamine; PS, phosphatidylserine; PA, phosphatidic acid; LPA, lysophosphatidic acid; PG, phosphatidylglycerol; PI, phosphatidylinositol.

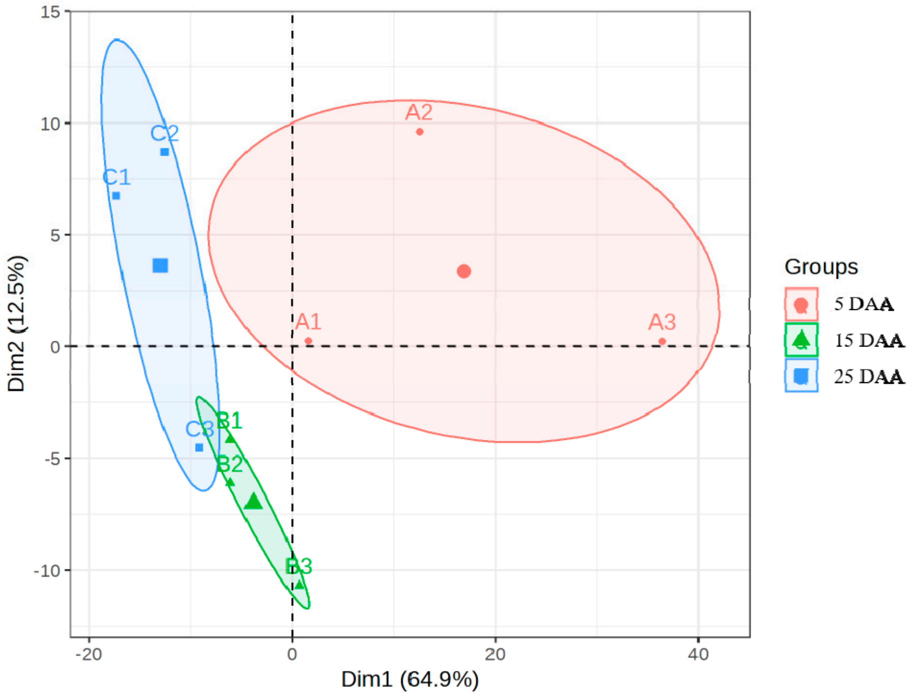

Figure S6. Principal component analysis (PCA) score plot of polar lipids classes in developing cotyledon of perilla. DAA indicates days after anthesis.
